# Patellofemoral alignment safe zones in robotic‐assisted total knee arthroplasty do not affect outcomes but do influence patellar resurfacing rates

**DOI:** 10.1002/ksa.12786

**Published:** 2025-07-18

**Authors:** Emanuele Diquattro, Luca Andriollo, Christos Koutserimpas, Jean Baltzer, Elvire Servien, Cécile Batailler, Sébastien Lustig

**Affiliations:** ^1^ Orthopaedics Surgery and Sports Medicine Department FIFA Medical Center of Excellence, Croix‐Rousse Hospital, Lyon University Hospital, Hospices Civils de Lyon Lyon France; ^2^ SC Ortopedia‐Traumatologia e Chirurgia Protesica e dei Reimpianti di Anca e Ginocchio, IRCCS Istituto Ortopedico Rizzoli Bologna Italy; ^3^ Sezione di Chirurgia Protesica ad Indirizzo Robotico ‐ Unità di Traumatologia Dello Sport, Ortopedia e Traumatologia, Fondazione Poliambulanza Istituto Ospedaliero Brescia Italy; ^4^ School of Rehabilitation Health Sciences University of Patras Patras Greece; ^5^ LIBM‐EA 7424, Interuniversity Laboratory of Biology of Mobility, Claude Bernard Lyon 1 University Lyon France; ^6^ Univ Lyon, Claude Bernard Lyon 1 University, IFSTTAR, LBMC UMR_T9406 Lyon France

**Keywords:** anterior compartment, functional alignment, patellofemoral joint, patellar resurfacing, patellar tracking, robotic‐assisted TKA

## Abstract

**Purpose:**

Anterior compartment management remains a challenging aspect of total knee arthroplasty (TKA), particularly in personalised alignment strategies. This study aimed to assess whether restoring patellofemoral alignment parameters within predefined safe zones—specifically patellar tilt (PTi), patellar translation (PTr) and patellar offset (PO)—is associated with improved clinical outcomes following robotic‐assisted TKA (rTKA).

**Methods:**

This retrospective study included 283 patients who underwent primary rTKA between March 2021 and January 2023 using functional alignment (FA) or functional knee positioning (FKP) principles. All surgeries were performed using a CT‐based robotic system (Mako, Stryker). Patients were stratified into groups based on radiographic values of PTi, PTr and ΔPO, using thresholds derived from prior robotic studies to define safe zones. Clinical outcomes at a mean follow‐up of 2.8 ± 0.8 years included Knee Society Score (KSS), Forgotten Joint Score‐12 (FJS‐12) and Kujala Anterior Knee Pain Scale (AKPS).

**Results:**

There were no statistically significant differences in final KSS, FJS‐12 or AKPS between groups within or outside the safe zones for PTi, PTr or ΔPO. However, patients with PTi < 0°, ΔPO > 0 mm (overstuffing) or PTr within ±2 mm showed significantly higher rates of patellar resurfacing (*p* < 0.001). No group demonstrated superior clinical outcomes across the three parameters studied.

**Conclusion:**

Restoring patellofemoral alignment parameters within predefined safe zones was not associated with improved short‐term clinical outcomes in rTKA. Robotic‐assisted FA provides accurate restoration of the anterior compartment, reducing reliance on patellar resurfacing in eligible patients. Our data suggest that target biomechanical parameters can potentially be achieved without resurfacing in cases where it is not indicated. Our hypothesis that patients within the proposed safe zones would demonstrate improved clinical outcomes was not supported by the current results. Further prospective studies are needed to determine whether femoral safe zones can predict long‐term benefit.

**Level of Evidence:**

Level II.

**Abbreviations**
AKPS: Anterior Knee Pain Scale
BMI: body mass index
CS: cruciate substitution
FA: functional alignment
FJS‐12: Forgotten Joint Score‐12
FKP: functional knee positioning
KA: kinematic alignment
KSS: Knee Society Score
MA: mechanical alignment
PO: patellar offset
PTi: patellar tilt
PTr: patellar translation
ROM: range of motion
rTKA: robotic‐assisted TKA
TKA: total knee arthroplasty

## INTRODUCTION

With the advancement of personalised surgical techniques, there is increasing interest in optimising the anterior knee compartment during total knee arthroplasty (TKA). Proper patellofemoral joint balance is critical to prevent anterior overstuffing and to enhance overall knee function [2, 8].

Despite progress in implant design, surgical methods, and assistive technologies, anterior knee pain, complications related to patellar prosthetic replacement and patient dissatisfaction remain common challenges [3, 12]. These issues highlight the need for a more tailored approach to anterior compartment management. The design of the prosthetic trochlea often differs from native anatomy, and conventional jig‐based instrumentation has limited ability to accurately restore the anterior compartment [4, 20].

Navigation and robotic‐assisted systems offer valuable tools for managing this so‐called 'third space' by enabling both detailed preoperative planning and dynamic intraoperative assessment [1, 26]. These systems enables real‐time feedback during planning, trialling and final implantation, helping to detect and correct risks of overstuffing or understuffing the anterior compartment. Such optimisation can improve intraoperative tracking and enhance the performance of the extensor mechanism post‐TKA [22].

Although patellar resurfacing has been investigated in earlier studies, data are lacking regarding its impact in rTKA performed using functional alignment (FA) or functional knee positioning (FKP) principles.

The primary aim of this study was to determine whether maintaining patellar alignment parameters within defined *safe zones*—specifically for patellar tilt (PTi), patellar translation (PTr) and patellar offset (PO)—results in improved clinical outcomes at final follow‐up. The secondary objective was to assess whether the clinical results within these *safe zones* differed between patients who underwent patellar resurfacing and those who did not during the rTKA procedure.

## METHODS

This study is a retrospective analysis of a prospectively maintained database. All patients who underwent primary robotic‐assisted total knee arthroplasty (rTKA) between March 2021 and January 2023, using principles of Functional Alignment (FA) or Functional Knee Positioning (FKP), were eligible for inclusion [21]. All procedures were performed using the Mako robotic‐arm‐assisted system (Stryker, Mako Surgical Corp.), and a minimum clinical follow‐up of 2 years was required.

Patients were excluded if preoperative, intraoperative, or follow‐up data were incomplete, or if the detailed robotic operative report was unavailable. Further exclusions included revision cases, prior femoral osteotomies, and post‐traumatic femoral osteoarthritis. After applying these criteria, 283 patients remained available for analysis at final follow‐up.

All patients received the Triathlon Total Knee System (Stryker, Mako Surgical Corp.). Patellar resurfacing was selectively performed in the presence of established anterior knee pain, patellofemoral joint osteoarthritis (Iwano grade ≥ 2), inflammatory or crystalline arthropathy, or intraoperative findings of excessive patellar tilt or maltracking [12]. In all cases requiring patellar resurfacing, a dome‐type component was implanted, consistent with the manufacturer‐supplied design specifically matched to the TKA system used.

Demographic variables such as age, sex, and body mass index (BMI) were recorded. The preoperative workup included knee range of motion (ROM) and the Knee Society Score (KSS). At final follow‐up, clinical outcomes were assessed using the KSS (knee and function sub‐scores), Forgotten Joint Score‐12 (FJS‐12), and the Kujala Anterior Knee Pain Scale (AKPS). ΔKSS knee and ΔKSS function were also calculated to capture the change from baseline.

Radiographic analysis was conducted using anteroposterior, lateral, Rosenberg, and skyline views, along with weight‐bearing full‐length standing X‐rays. Preoperative patellar offset (versus tilt/translation) was considered exclusively for postoperative comparison because it better defines the anatomy of the anterior/trochlear compartment and conventional alignment parameters are rendered unreliable by arthritic deformity, as they represent pathologic compensation rather than normal anatomy.

The patellar offset pre‐op and post‐op [14], the patellar translation post‐op [24], and the patellar tilt post‐op [7], were measured using standardised and documented techniques. The following anterior compartment parameters were measured:

- Patellar Tilt (PTi) in degrees (positive = medial opening, negative = lateral opening),
- Patellar Translation (PTr) in millimetres (positive = medial translation, negative = lateral translation),
- Patellar Offset (PO), defined as the anteroposterior patellar position relative to the femoral trochlea, and expressed as ΔPO = postoperative PO – preoperative PO.

The measurements demonstrated excellent inter‐observer reliability, with intraclass correlation coefficients (ICCs) of 0.91 for PTi, 0.92 for PTr and 0.90 for PO.

### Definition of safe zones

To objectively assess anterior compartment restoration, *safe zones* were defined for each parameter, based on previous literature using robotic datasets [9]. These thresholds represent the range within which the patellofemoral biomechanics are considered physiologically neutral and unlikely to result in overstuffing, maltracking or extensor mechanism dysfunction.

- PTi safe zone: 0°–5° medial opening
  ◦ Group A: PTi < 0° (lateral opening)
  ◦ Group B: PTi 0°–5° (safe zone)
  ◦ Group C: PTi > 5° (excessive medial tilt)
- PTr safe zone: –2 mm to +2 mm
  ◦ Group D: PTr < –2 mm (lateral translation)
  ◦ Group E: PTr –2 mm to + 2 mm (safe zone)
  ◦ Group F: PTr > +2 mm (medial translation)
- ΔPO safe zone: 0 mm to –5 mm (no anterior overstuffing, mild reduction acceptable)
  ◦ Group G: ΔPO > 0 mm (anterior overstuffing)
  ◦ Group H: ΔPO 0 mm to –5 mm (safe zone)
  ◦ Group I: ΔPO < –5 mm (anterior understuffing)

The FA and FKP techniques aim to replicate native trochlear geometry [21], and the use of image‐based robotic systems—with accuracy below 1 mm and 1° [5, 23] —permits precise measurement and correction of these parameters intraoperatively.

### Ethical Approval

This study was conducted in accordance with the ethical standards of the 1964 Declaration of Helsinki and complied with HIPAA regulations. It adhered to the MR004 Reference Methodology of the French Commission Nationale de l'Informatique et des Libertés (Ref. 2229975V0), and all patients provided written informed consent.

### Statistical analysis

Continuous variables are expressed as mean ± standard deviation (SD), and categorical variables as frequencies and percentages. Data normality was assessed using the Shapiro–Wilk test.

Comparisons between groups were made using one‐way ANOVA for normally distributed continuous variables, and the Kruskal–Wallis test otherwise. Chi‐square tests were used for categorical variables. Post hoc comparisons employed Bonferroni correction where appropriate. A *p*‐value < 0.05 was considered statistically significant. Analyses were conducted using Python v3.11 (Python Software Foundation) and the *statsmodels* library (v0.13).

## RESULTS

Out of the 283 patients included, 151 (53.4%) underwent patellar resurfacing, while 132 (46.6%) retained their native patella. The mean follow‐up duration was 2.8 ± 0.8 years. The overall mean values of the anterior compartment parameters were:

- PTi: 3.6° ± 5.4° (medial opening),
- PTr: –2.8 ± 4.8 mm (lateral translation),
- PO : 30.5 ± 3.9 mm,
- Postoperative PO: 26.8 ± 3.2 mm,
- ΔPO: –3.7 ± 3.1 mm (reduction in PO, reflecting a decreased anterior offset).

### PTi—Safe zone analysis

**Table 1:** Preoperative and final follow‐up data of Group A, Group B and Group C categorised based on PTi (patellar tilt).

Preoperative data				
	Group A (*N* = 60)	Group B (*N* = 111)	Group C (*N* = 112)	*p*‐Value
Age (years)	68.7 (SD 7.5)	67.2 (SD 8.0)	69.3 (SD 8.5)	0.17
Sex (female)	47 (78.3%)	58 (52.2%)	56 (50.0%)	**<0.001**
BMI (kg/m²)	27.8 (SD 4.7)	28.3 (SD 5.0)	29.1 (SD 4.7)	0.15
KSS knee	65.7 (SD 11.6)	63.5 (SD 12.8)	65.1 (SD 13.8)	0.52
KSS function	68.8 (SD 13.1)	66.5 (SD 16.6)	68.9 (SD 15.9)	0.49
Recurvatum (degrees)	0.5 (SD 1.8)	0.7 (SD 2.7)	0.4 (SD 1.8)	0.52
Flexion contracture (degrees)	2.3 (SD 3.8)	2.1 (SD 3.9)	2.5 (SD 4.5)	0.81
Maximum flexion (degrees)	117.4 (SD 11.4)	119.4 (SD 12.0)	120.1 (SD 12.0)	0.32
Patellar resurfacing (yes)	54 (90.0%)	58 (52.3%)	39 (34.8%)	**<0.001**
Liner (CS)	21 (35.0%)	38 (34.2%)	38 (33.9%)	0.99
Final follow‐up data				
KSS knee	92.3 (SD 7.3)	93.0 (SD 8.2)	92.8 (SD 9.3)	0.87
KSS function	91.4 (SD 9.3)	92.0 (SD 10.2)	91.8 (SD 9.4)	0.92
Recurvatum (degrees)	1.0 (SD 2.2)	1.4 (SD 2.4)	1.0 (SD 2.5)	0.31
Flexion contracture (degrees)	0.1 (SD 0.8)	0.3 (SD 1.4)	0.2 (SD 1.2)	0.53
Maximum flexion (degrees)	123.6 (SD 9.8)	125.6 (SD 9.9)	124.1 (SD 10.9)	0.39
FJS‐12	78.9 (SD 19.7)	76.4 (SD 23.1)	73.5 (SD 24.2)	0.30
AKPS	89.7 (SD 12.0)	90.1 (SD 12.3)	86.4 (SD 17.2)	0.13

*Note*: Group A includes patients with PTi < 0° (lateral opening), Group B includes patients with PTi between 0° and 5°, and Group C includes patients with PTi > 5° (medial opening).

Abbreviations: AKPS, Kujala Anterior Knee Pain Scale; FJS‐12, Forgotten Joint Score; KSS, Knee Society Score; SD, standard deviation.

**Figure 1:**
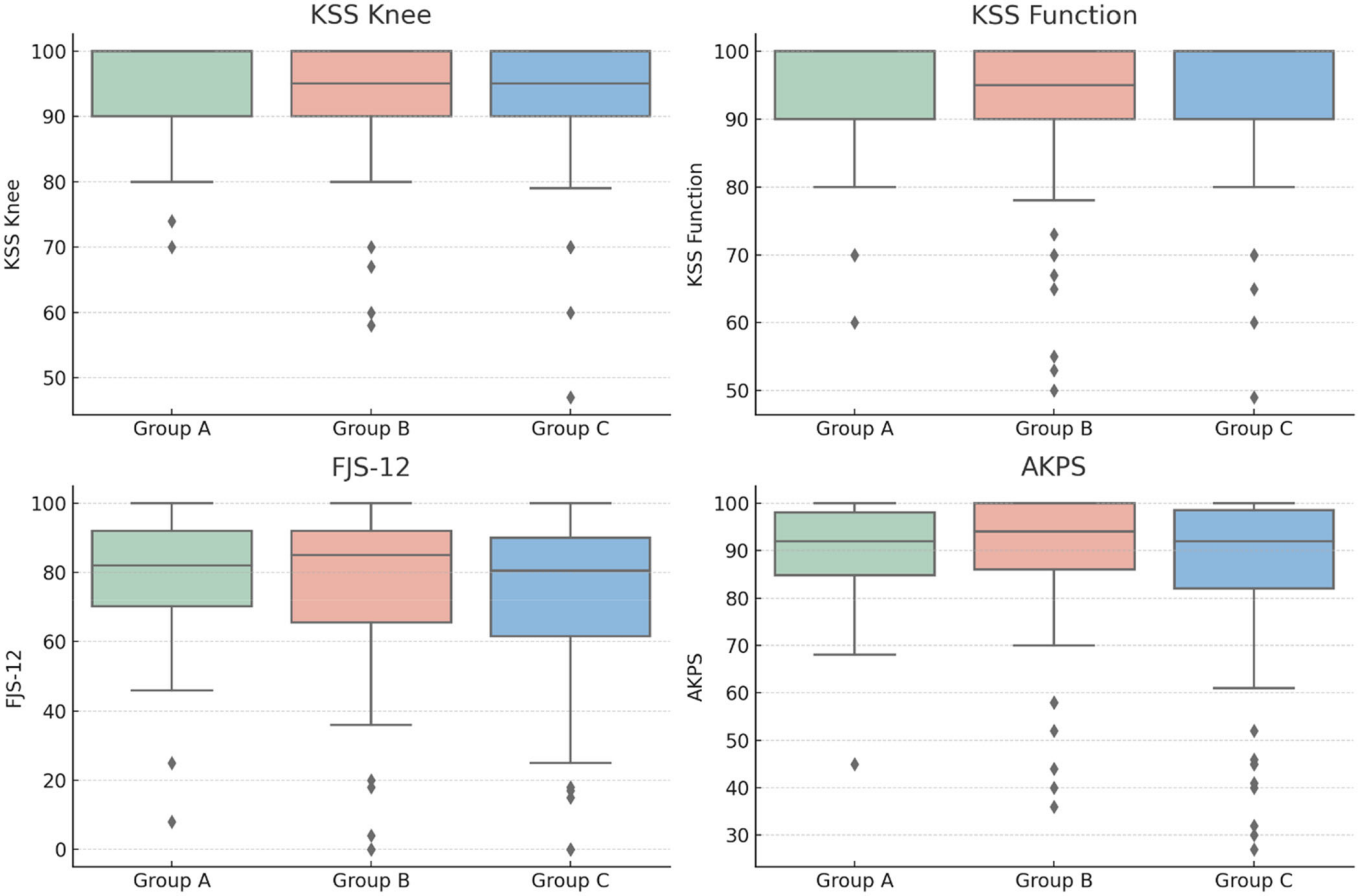
Graphical representation using box plots of the parameters analysed in the comparison between groups, categorised based on patellar tilt (PTi), which represents the tilt angle of the patella. Based on PTi, patients were classified into Group A (PTi < 0°), Group B (PTi between 0° and 5°), and Group C (PTi > 5°). AKPS, Kujala Anterior Knee Pain Scale; FJS‐12, Forgotten Joint Score; KSS, Knee Society Score.

Patients were divided into three groups (Table 1 and Figure 1):

- Group A: PTi < 0° (lateral tilt), *n* = 60 (21.2%)
- Group B: PTi 0°–5° (safe zone), *n* = 111 (39.2%)
- Group C: PTi > 5° (excessive medial tilt), *n* = 112 (39.6%).

There were no significant differences in baseline demographic or clinical parameters (age, BMI, KSS preop scores, ROM, recurvatum and flexion contracture). The use of CS liners was similar across groups.

However, a significant difference in patellar resurfacing rates was observed (*p* < 0.001), with the highest prevalence in Group A (tilt < 0°). Pairwise comparisons showed significant differences between:

- Group A versus Group B (*p* < 0.001),
- Group A versus Group C (*p* < 0.001),
- Group B versus Group C (*p* = 0.013).

At final follow‐up, no significant differences were found between groups regarding:

- KSS knee or function,
- Maximum flexion,
- FJS‐12 or AKPS.

### PTr—Safe zone analysis

**Table 2:** Preoperative and final follow‐up data of Group D, Group E and Group F categorised based on PTr (patellar translation).

Preoperative data				
	Group D (*N* = 147)	Group E (*N* = 108)	Group F (*N* = 28)	*p*‐Value
Age (years)	68.2 (SD 8.7)	69.4 (SD 7.2)	65.5 (SD 7.9)	0.07
Sex (female)	74 (50.3%)	67 (62.0%)	16 (57.1%)	0.18
BMI (kg/m²)	29.1 (SD 5.1)	27.6 (SD 4.5)	29.2 (SD 4.6)	0.06
KSS knee	64.3 (SD 13.5)	65.7 (SD 12.9)	62.4 (SD 9.1)	0.43
KSS function	68.2 (SD 16.7)	69.4 (SD 14.4)	62.2 (12.5)	0.09
Recurvatum (degrees)	0.4 (SD 1.8)	0.7 (SD 2.5)	0.7 (SD 2.2)	0.65
Flexion contracture (degrees)	2.5 (SD 4.4)	2.2 (SD 3.9)	1.6 (SD 3.1)	0.54
Maximum flexion (degrees)	119.0 (SD 12.1)	119.6 (SD 11.9)	118.4 (SD 10.2)	0.87
Patellar resurfacing (yes)	43 (29.3%)	93 (86.1%)	15 (53.6%)	**<0.001**
Liner (CS)	53 (36.0%)	40 (37.0%)	4 (14.3%)	0.14
Final follow‐up data				
KSS knee	93.6 (SD 8.4)	92.0 (SD 7.6)	91.2 (SD 11.5)	0.21
KSS function	92.4 (SD 8.8)	91.5 (SD 8.8)	89.8 (SD 15.6)	0.41
Recurvatum (degrees)	1.3 (SD 2.5)	0.8 (SD 1.9)	1.6 (SD 3.1)	0.21
Flexion contracture (degrees)	0.2 (SD 1.1)	0.2 (SD 1.1)	0.6 (SD 1.6)	0.23
Maximum flexion (degrees)	124.4 (SD 10.7)	124.0 (SD 10.6)	127.6 (SD 6.3)	0.27
FJS‐12	75.0 (SD 22.7)	77.7 (SD 21.7)	73.3 (SD 27.4)	0.52
AKPS	87.6 (SD 15.1)	90.1 (SD 12.0)	87.7 (SD 18.7)	0.38

*Note*: Group D includes patients with PTr < −2 mm (lateral translation), Group E includes patients with PTr between −2 mm and 2 mm (neutral zone), and Group F includes patients with PTr > 2 mm (medial translation).

Abbreviations: AKPS, Kujala Anterior Knee Pain Scale; FJS‐12, Forgotten Joint Score; KSS, Knee Society Score; SD, standard deviation.

**Figure 2:**
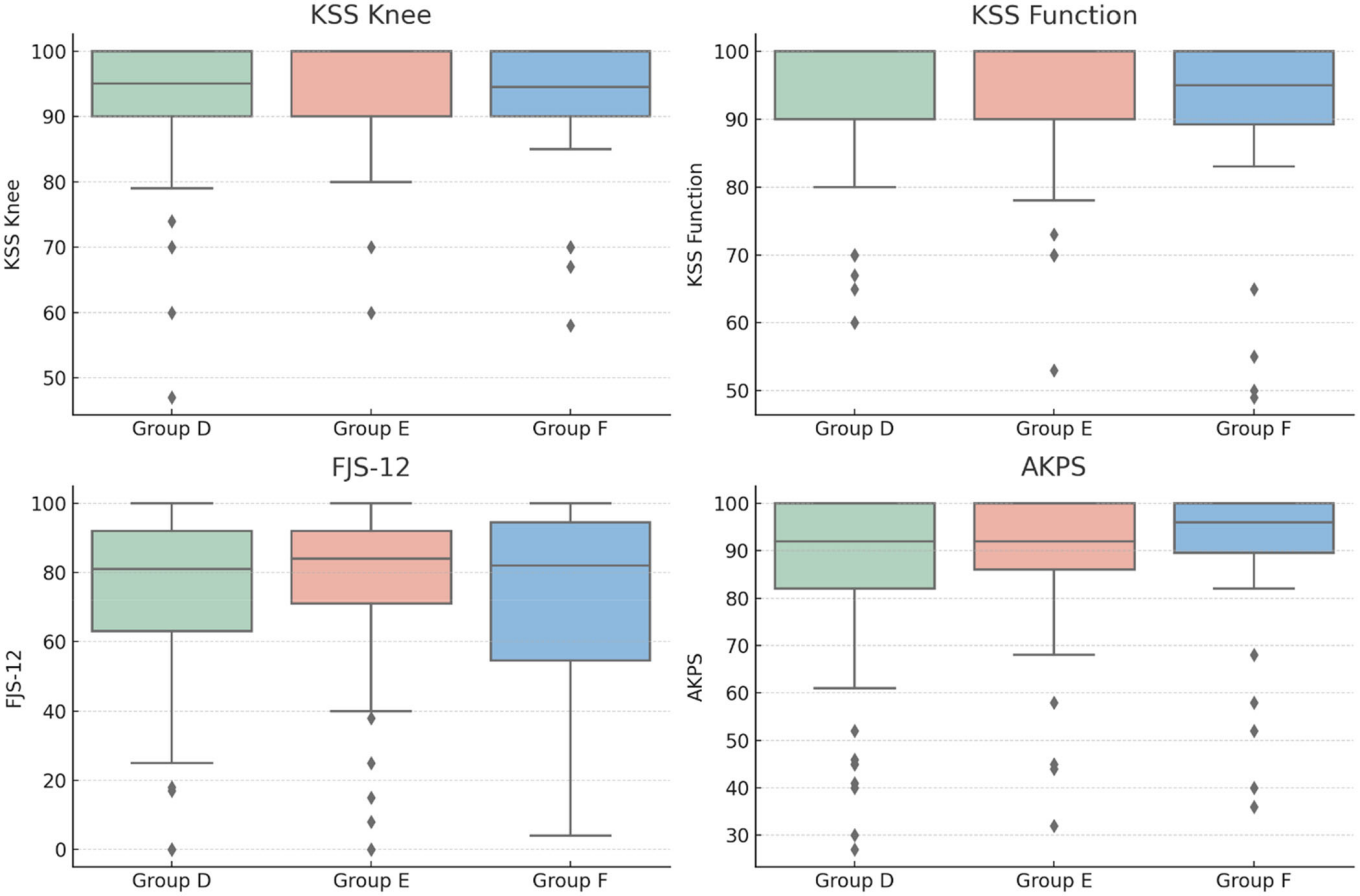
Graphical representation using box plots of the parameters analysed in the comparison between groups, categorised based on patellar translation (PTr), which represents the translation of the patella. Based on PTr, patients were classified into Group D (PTr < −2 mm, lateral translation), Group E (PTr between −2 mm and 2 mm, neutral zone), and Group F (PTr > 2 mm, medial translation). AKPS, Kujala Anterior Knee Pain Scale; FJS‐12, Forgotten Joint Score; KSS, Knee Society Score.

Groups based on postoperative PTr (Table 2 and Figure 2):

- Group D: PTr < –2 mm (lateral translation), *n* = 147 (51.9%),
- Group E: PTr –2 mm to + 2 mm (safe zone), *n* = 108 (38.2%),
- Group F: PTr > +2 mm (medial translation), *n* = 28 (9.9%).

There were no significant differences in age, sex, BMI, preoperative KSS, ROM or use of CS liners across groups.

A statistically significant variation was noted in patellar resurfacing rates (*p* < 0.001), with:

- Group E (safe zone): highest rate (86.1%),
- Group F: 53.6%,
- Group D: 29.3%.

Significant differences were found between:

- Group D versus Group E (*p* < 0.001),
- Group E versus Group F (*p* < 0.001),
- Group D versus Group F (*p* = 0.149; not significant).

No significant differences were observed in any final follow‐up functional scores.

### ΔPO—Safe zone analysis

**Table 3:** Preoperative and final follow‐up data of Group G, Group H, and Group I categorised based on ΔPO (difference between pre‐ and postoperative measurements of patellar offset).

Preoperative data				
	Group G (*N* = 33)	Group H (*N* = 160)	Group I (*N* = 90)	*p*‐Value
Age (years)	68.8 (SD 6.8)	68.6 (SD 8.1)	67.9 (SD 8.7)	0.74
Sex (female)	20 (60.6%)	100 (62.5%)	41 (45.6%)	**0.005**
BMI (kg/m²)	30.1 (SD 4.6)	28.9 (SD 5.0)	27.3 (SD 4.3)	**0.006**
KSS knee	65.6 (SD 14.9)	64.1 (SD 12.5)	65.3 (SD 12.9)	0.72
KSS function	68.4 (SD 17.4)	68.3 (SD 14.5)	67.4 (SD 16.6)	0.90
Recurvatum (degrees)	0.8 (SD 1.8)	0.6 (SD 2.3)	0.4 (SD 1.9)	0.76
Flexion contracture (degrees)	3.2 (SD 5.4)	2.2 (SD 3.9)	2.1 (SD 3.9)	0.41
Maximum flexion (degrees)	118.2 (SD 12.0)	119.0 (SD 11.6)	119.8 (SD 12.3)	0.78
Patellar resurfacing (yes)	28 (84.8%)	84 (52.5%)	39 (43.3%)	**<0.001**
Liner (CS)	12 (36.4%)	55 (34.4%)	30 (33.3%)	0.95
Final follow‐up data				
KSS knee	93.8 (SD 6.5)	92.3 (SD 9.1)	93.1 (SD 8.0)	0.60
KSS function	92.3 (SD 7.9)	91.7 (SD 10.1)	91.8 (SD 9.6)	0.96
Recurvatum (degrees)	0.9 (SD 2.3)	1.1 (SD 2.2)	1.2 (SD 2.6)	0.83
Flexion contracture (degrees)	0.3 (SD 1.2)	0.2 (SD 1.2)	0.3 (SD 1.1)	0.81
Maximum flexion (degrees)	124.1 (SD 8.6)	124.5 (SD 10.5)	124.8 (SD 10.6)	0.95
FJS‐12	83.6 (SD 15.3)	73.8 (SD 24.4)	76.5 (SD 21.6)	0.07
AKPS	92.9 (SD 7.9)	86.7 (SD 15.6)	90.2 (SD 13.5)	0.03

*Note*: Group G includes patients with ΔPO > 0 mm (overstuffing), Group H includes patients with ΔPO between 0 mm and –5 mm (moderate anterior compartment), and Group I includes patients with ΔPO < –5 mm (significant anterior understuffing).

Abbreviations: AKPS, Kujala Anterior Knee Pain Scale; FJS‐12, Forgotten Joint Score; KSS, Knee Society Score; SD, standard deviation.

**Figure 3:**
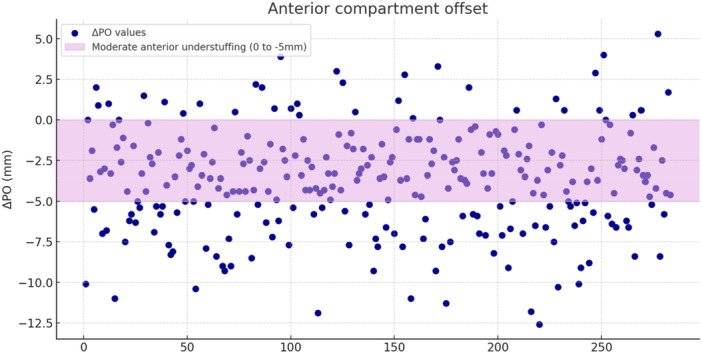
Distribution of the variation in patellar offset between preoperative and postoperative, indicated as ΔPO.

**Figure 4:**
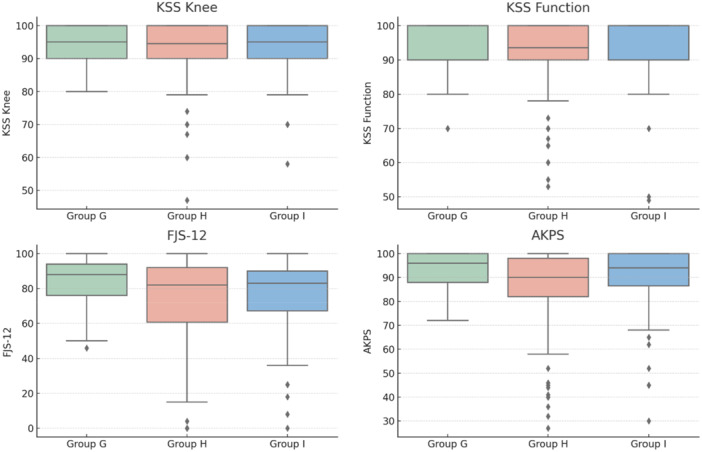
Graphical representation using box plots of the parameters analysed in the comparison between groups, categorised based on ΔPO, which represents the difference between preoperative and postoperative measurements. Based on ΔPO, patients were classified into Group G (ΔPO > 0 mm, overstuffing), Group H (ΔPO between 0 mm and –5 mm, moderate anterior compartment), and Group I (ΔPO < –5 mm, significant anterior understuffing). AKPS, Kujala Anterior Knee Pain Scale; FJS‐12, Forgotten Joint Score; KSS, Knee Society Score.

Groups based on change in PO (Table 3 and Figures 3 and 4):

- Group G: ΔPO > 0 mm (anterior overstuffing), *n* = 33 (11.7%),
- Group H: ΔPO 0 mm to –5 mm (safe zone), *n* = 160 (56.5%),
- Group I: ΔPO < –5 mm (understuffing), *n* = 90 (31.8%).

No significant differences were found across groups for age, preoperative KSS scores, ROM parameters, or liner usage. However:

- Sex distribution varied significantly (*p* = 0.005), with Group H (safe zone) showing the highest proportion of female patients (62.5%).

### **Patellar resurfacing rates** differed significantly (*p* < 0.001)

- Group G (overstuffing): 84.8%,
- Group H (safe zone): 52.5%,
- Group I (understuffing): 43.3%.

Significant differences were observed between:

- Group G versus Group H (*p* = 0.001),
- Group G versus Group I (*p* < 0.0001),
- Group H versus Group I (not significant, *p* = 0.2077).

At final follow‐up:

- No significant differences in KSS, ROM or FJS‐12 scores.
- AKPS scores showed a significant group effect (*p* = 0.03):
  ◦ Group G: 92.9 ± 7.9,
  ◦ Group I: 90.2 ± 13.5,
  ◦ Group H: 86.7 ± 15.6.

However, post hoc comparisons did not reach statistical significance:

- G versus H (*p* = 0.06, trending),
- G versus I (*p* = 0.63),
- H versus I (*p* = 0.14).

## DISCUSSION

The primary findings of this study suggest that maintaining patellofemoral alignment parameters within the proposed *safe zones*—for PTi, PTr and ΔPO—was not associated with significant differences in clinical outcomes at a mean follow‐up of nearly three years. Nevertheless, distinct patterns emerged regarding the association between specific patellofemoral alignment profiles and the frequency of patellar resurfacing.

Specifically, patients with increased postoperative patellar offset (Group G), lateral patellar tilt (Group A, PTi < 0°), or neutral translation (Group E, –2 mm < PTr < 2 mm) demonstrated significantly higher rates of patellar resurfacing. These findings suggest that patellar resurfacing may influence patellofemoral alignment parameters, even though its impact on mid‐term functional scores appears limited.

Despite the lack of a clear clinical advantage for remaining within *safe zones*, the concept remains relevant in the context of robotic‐assisted personalised surgery. While traditional TKA focuses primarily on tibiofemoral alignment, robotic systems enable precise assessment of the anterior compartment—a dimension often overlooked in mechanical alignment (MA) techniques [10]. Previous studies have shown that MA and conventional implants do not adequately replicate native trochlear anatomy, leading to patellofemoral incongruencies [19].

In contrast, robotic systems incorporating preoperative CT allow the femoral implant to be virtually positioned in alignment with the native trochlear groove, thus improving patellar tracking and extensor mechanism kinematics [11, 15, 18]. While patellar resurfacing remains a non‐robotic procedure, the robotic system enables precise adjustment of the femoral trochlear component. Our surgical technique prioritises preservation of native trochlear anatomy to optimise gap balancing across flexion‐extension while achieving target coronal alignment. Unlike trochlear offset restoration—previously well‐documented—patellar outcomes can only be evaluated postoperatively through radiographic assessment of the integrated reconstruction. However, intraoperative robotic‐assisted evaluation for maltracking remains crucial, as supported by existing literature [18]. Among alignment strategies, functional alignment (FA) appears to strike a better balance: it exceeds safety thresholds for implant rotation and coronal alignment less frequently than both MA and kinematic alignment (KA) [11, 25].

Our study confirms the potential of image‐based robotic systems to optimise patellofemoral relationships, even in the absence of resurfacing. The subvastus approach further contributes to patellar stability, potentially allowing for moderate understuffing without compromising function. The absence of significant functional outcome differences across groups supports previous observations that patellofemoral kinematics can be successfully restored using FA principles, especially when combined with robotic guidance [6, 16].

When focusing on PTi, patients with lateral tilt (<0°) had higher resurfacing rates, especially among females. Similarly, neutral PTr (–2 mm to 2 mm) was associated with a higher resurfacing frequency. These associations may reflect a surgical preference to resurface the patella in borderline or ambiguous tracking situations, rather than a causal relationship. For ΔPO, the overstuffed group (ΔPO > 0 mm) showed significantly more resurfacing compared to both the safe zone (ΔPO 0 to –5 mm) and understuffed group (ΔPO < –5 mm).

Interestingly, although differences in anterior compartment restoration were observed radiographically, they did not translate into significant differences in functional outcomes such as KSS, FJS‐12 or AKPS at 2 years. Only a non‐significant trend toward improved AKPS was seen in the overstuffed group. These findings are in line with prior literature suggesting that clinical scores may not directly correlate with radiographic alignment metrics [14, 17].

Previous robotic studies have shown that FA can result in global anterior compartment *understuffing* in extension and mid‐flexion, while overstuffing can occur at 90°, potentially leading to anterior pain or reduced flexion [9]. Maintaining ΔPO within a moderate range may therefore help prevent overstuffing‐related complications, especially in high flexion.

Finally, it is worth noting that radiographic markers such as increased lateral PTr and high PTi have been associated with anterior pain and flexion stiffness post‐TKA [2, 13]. Although our findings did not confirm a direct link, they reinforce the potential role of radiographic alignment as a marker of anterior compartment health.

### Limitations

This study has several limitations. First, the mean follow‐up duration was relatively short, although sufficient to capture postoperative anterior pain and early functional outcomes. Continued follow‐up will be critical for assessing long‐term progression. Second, the retrospective nature and single‐center setting may limit generalisability, despite the prospectively maintained data. Third, only one implant design was studied. While this ensured consistency, future research should include various designs, given the known variability in trochlear geometry. Also, while we used ΔPO to characterise anterior compartment restoration, integrating this with tilt and translation into a composite score might enhance sensitivity. Lastly, No power analysis was conducted for this study.

## CONCLUSION

While the proposed patellofemoral safe zones did not demonstrate short‐term clinical benefits in this study, they may still serve as useful intraoperative references in robotic‐assisted TKA. Our results tentatively suggest that precise anterior compartment restoration through functional alignment principles could potentially decrease reliance on patellar resurfacing, though this observation requires careful interpretation. The relationship between radiographic alignment parameters and clinical outcomes merits further investigation through well‐designed prospective studies with extended follow‐up periods to properly assess the value of personalised patellofemoral alignment strategies.

## AUTHOR CONTRIBUTIONS

**Emanuele Diquattro**: Conceptualisation; methodology; data curation; writing an original draft. **Luca Andriollo**: Conceptualisation; methodology; data curation; writing an original draft. **Christos Koutserimpas**: Data curation; methodology writing; reviewing and editing. **Jean Baltzer**: Data curation; methodology writing. **Elvire Servien**: Conceptualisation; methodology; writing; reviewing. **Cécile Batailler**: Conceptualisation; supervision; validation; writing; reviewing and editing. **Sébastien Lustig:** Conceptualisation; supervision; validation; writing; reviewing and editing.

## CONFLICTS OF INTEREST STATEMENT

Emanuele Diquattro, Luca Andriollo, Christos Koutserimpas and Jean Baltzer have nothing to declare. Elvire Servien: Consultant for Corin. Institutional research support from Corin, Amplitude. Cécile Batailler: Consultant for Stryker, Smith Nephew and Groupe Lepine. Sébastien Lustig: Royalties from Smith Nephew, Stryker and Serf. Consultant for Stryker, Heraeus; Institutional research support from Amplitude and Groupe Lepine; Editorial Board for *Journal of Bone and Joint Surgery* (Am).

## ETHICS STATEMENT

The study has been approved by the scientific committee of Hospices Civils de Lyon, France. It adhered to the MR004 Reference Methodology of the French Commission Nationale de l'Informatique et des Libertés (Ref. 2229975V0). All patients provided written informed consent.
